# Modeling the Posture–Movement Continuum: Predictive Mapping of Spinopelvic Control Across Gait Speeds

**DOI:** 10.3390/jcm15010073

**Published:** 2025-12-22

**Authors:** Rofaida Mohamed Elsayed, Ibrahim M. Moustafa, Abdulla Alrahoomi, Mishal M. Aldaihan, Abdulrahman M. Alsubiheen, Iman Akef Khowailed

**Affiliations:** 1 Department of Physiotherapy, College of Health Sciences, University of Sharjah, Sharjah 27272, United Arab Emiratesiabuamr@sharjah.ac.ae (I.M.M.); 2 Neuromusculoskeletal Rehabilitation Research Group, RIMHS–Research Institute of Medical and Health Sciences, University of Sharjah, Sharjah 27272, United Arab Emirates; 3 Faculty of Physical Therapy, Cairo University, Giza 12613, Egypt; 4 Sheikh Tahnoon Medical City Rehabilitation Hospital, Al Ain, United Arab Emirates; 5 Department of Rehabilitation Health Sciences, College of Applied Medical Sciences, King Saud University, P.O. Box 10219, Riyadh 11433, Saudi Arabia; mishaldaihan@ksu.edu.sa

**Keywords:** forward head posture, quadriceps angle, posture, gait, spinal balance

## Abstract

**Background**: This study investigated how static postural parameters influence dynamic spinopelvic balance across varying walking speeds. One hundred healthy young adults (aged 18–25) underwent rasterstereographic assessment (DIERS 4Dmotion^®^) to quantify static global alignment metrics including craniovertebral angle (CVA), Q-angle, sagittal and coronal imbalance, pelvic rotation, torsion, obliquity, vertebral rotation, thoracic kyphosis, lumbar lordosis, and pelvic tilt, followed by dynamic spinopelvic analysis during treadmill walking at 1, 2, 4, and 5 km/h. **Methods**: Multiple linear regression models were used to determine the predictive value of static postural measures for dynamic outcomes at each speed. At slower walking speeds (1–2 km/h), static alignment variables significantly predicted dynamic spinopelvic parameters (adjusted R^2^ = 0.53–0.73; RMSE = 0.59–0.81), with CVA, sagittal imbalance, and pelvic torsion emerging as the most consistent predictors. **Results**: At higher speeds (4–5 km/h), predictive strength declined substantially (adjusted R^2^ = 0.04–0.34), indicating a shift from posture-driven to neuromuscular-governed gait control. The Q-angle showed limited and inconsistent predictive value across all conditions. **Conclusions**: Overall, static postural alignment, particularly CVA, sagittal imbalance, and pelvic torsion, serves as a moderate predictor of spinopelvic dynamics at slow to moderate gait speeds but loses explanatory power as velocity increases, emphasizing the growing role of neuromuscular control in maintaining dynamic balance. These findings highlight the clinical relevance of integrating both static and dynamic assessments to comprehensively evaluate postural and locomotor function.

## 1. Introduction

The static global alignment of the spine pelvis lower limb complex, encompassing sagittal imbalance (anterior–posterior trunk shift), coronal imbalance (lateral deviation in the frontal plane), pelvic rotation, pelvic torsion, pelvic obliquity, vertebral rotation, thoracic kyphosis, lumbar lordosis, pelvic tilt, craniovertebral angle (CVA), and Quadriceps angle (Q angle), plays a fundamental role in maintaining postural equilibrium and optimizing dynamic movement [1,2,3]. Deviations in these alignment parameters alter the spatial relationship between body segments and can profoundly affect the mechanical function of the spine and pelvis during functional tasks such as gait [4,5,6]. When the spatial configuration of these segments is disturbed, the center of mass shifts forward or laterally, prompting compensatory adjustments throughout the trunk and pelvis. These compensations, which may manifest as increased thoracic kyphosis, flattened lumbar lordosis, or abnormal pelvic orientation; disrupt intersegmental coordination; and compromise load distribution during locomotion [7,8,9]. Such alterations do not represent isolated postural faults but rather systemic changes in global alignment that affect mechanical efficiency and stability across multiple body regions [10,11]. The consequences of static global misalignment extend beyond structural compensation. They also affect sensorimotor control and neuromuscular coordination. Abnormal alignment modifies joint loading and soft tissue tension, leading to altered afferent input from muscle and joint mechanoreceptors, particularly in the upper cervical spine (C1–C3), which is densely populated with proprioceptors essential for spatial orientation and postural reflexes [12,13]. Distorted sensory input can impair the integration of cervicovestibular and cervicoocular reflexes, diminishing the central nervous system’s capacity to coordinate head, trunk, and pelvic motion during dynamic activities [14]. This disruption contributes to cervical joint position errors, delayed neuromuscular responses, and balance deficits [15] and over time, may reinforce maladaptive movement strategies that increase spinal loading and perpetuate instability [16].

The biomechanical implications of static global alignment deviations become particularly evident during locomotion, a complex motor task governed by both feedback and feedforward control mechanisms. At slower walking speeds, feedback-driven adjustments relying on proprioceptive and visual inputs dominate. In contrast, higher speeds increasingly engage predictive, feedforward strategies and preprogrammed central pattern generator activity, imposing greater demands on neuromuscular coordination and segmental control [17,18]. As gait velocity rises, ground reaction forces, multiplanar loading, and coordination complexity all increase [19,20]. Although static standing postural measures do not fully capture the spine–pelvis configuration during walking, longitudinal and repeated measure studies indicate that static alignment parameters are associated with concurrent or subsequent changes in gait and balance. For instance, static posturography scores have been shown to predict changes in walking speed and trunk inclination during locomotion, and standing sagittal alignment often correlates with dynamic pelvic orientation and trunk posture in healthy adults [21,22,23]. These transitions intensify the biomechanical and neural challenges associated with maintaining spinopelvic stability. Despite this, little is known about how static global alignment parameters influence speed dependent control strategies or how they predict spinopelvic responses under varying locomotor demands.

Previous investigations have focused on gait adaptations in specialized populations, such as individuals with traumatic amputations [24], yet systematic evidence is lacking on how static global alignment parameters including CVA, Q-angle, and pelvic alignment measures predict dynamic spinopelvic behavior in healthy individuals across different walking speeds. Malalignment may impair thoracolumbar dissociation, alter trunk pelvis coupling, and delay activation of deep stabilizers such as the transversus abdominis and multifidus [25]. These impairments may give rise to compensatory gait strategies such as shortened stride length, excessive lateral sway, or asymmetric weight shifting, which may become more pronounced during high-speed gait, when predictive motor control predominates [26,27]. Furthermore, postural deviations have been linked to increased spinal loading and delayed proprioceptive processing [28,29,30], but their predictive contribution to dynamic spinopelvic behavior across velocity dependent conditions remain unclear. Accordingly, this study aimed to determine whether static global alignment parameters including CVA, Q-angle, and spinopelvic orientation measures predict dynamic spinopelvic balance across different walking speeds. By applying a predictive modeling framework, this study seeks to identify the most influential biomechanical markers of gait related imbalance.

## 2. Materials and Methods

### 2.1. Study Design and Sampling

This cross-sectional predictive modeling study was conducted at the Biomechanics and Rehabilitation Research Laboratory, University of Sharjah, Sharjah, United Arab Emirates. The study was approved by the Research Ethics Committee of the University of Sharjah (REC-24-12-24-02-PG), and all participants provided written informed consent prior to enrollment. A total of 100 healthy young adults, aged 18–25 years, were recruited through convenience sampling from the university’s student and staff population. Participants were excluded if they had persistent musculoskeletal pain lasting more than two consecutive days in the preceding six months; any spinal or extremity deformities; inflammatory joint disease; history of musculoskeletal surgery within the past year; neurological or vestibular disorders; body mass index (BMI) greater than 30 kg/m^2^ (to reduce potential confounding effects of obesity on posture and gait); or pregnancy. Eligibility was confirmed prior to data collection using demographic and health questionnaires, anthropometric assessment, and static posture evaluation performed with rasterstereographic analysis.

### 2.2. Outcome Measures

Static and dynamic spinopelvic parameters were assessed using rasterstereographic analysis with the DIERS Formetric 4D Motion^®^ system (DIERS International GmbH, Schlangenbad, Germany; software version (v3.17.0.27)), a validated, non-invasive optical system that reconstructs three-dimensional spinal and pelvic alignment without radiation exposure [31,32]. In addition, static outcome measures including CVA and Q-angle were measured using the PostureScreen^®^ Mobile application (PSM; PostureCo Inc., Atlanta, GA, USA; software version 15.2.25).

#### 2.2.1. Static Parameters

**Figure 1:**
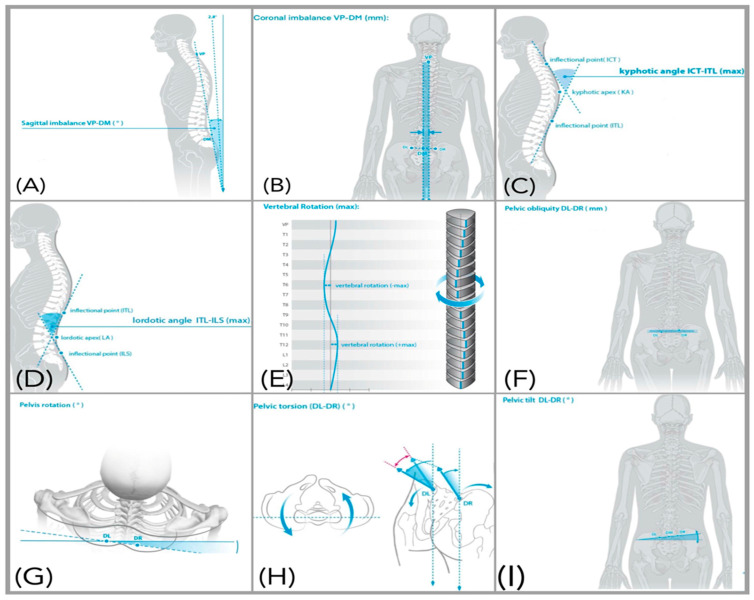
Rasterstereographic visualization of spino-pelvic alignment parameters: (**A**) Sagittal imbalance, (**B**) Coronal imbalance, (**C**) Kyphotic angle, (**D**) Lordotic angle, (**E**) Vertebral rotation, (**F**) Pelvic obliquity, (**G**) Pelvic rotation, (**H**) Pelvic torsion, (**I**) Pelvic tilt. Dashed lines represent reference axes and angular baselines used for parameter calculation. With permission from DIERS Formetric 4D^®^ (DIERS International GmbH, Schlangenbad, Germany).

Static spinopelvic parameters were measured under quiet standing conditions and included sagittal imbalance (anterior–posterior trunk shift), coronal imbalance (lateral deviation in the frontal plane), pelvic rotation, pelvic torsion, pelvic obliquity, vertebral rotation, thoracic kyphosis angle, lumbar lordosis angle, and pelvic tilt (Table 1 and Figure 1). In addition, CVA represents forward head posture and is calculated as the angle between a horizontal line through C7 and a line connecting C7 to the tragus (lower values indicate greater forward head posture) [33], and Q-angle defined as the angle formed between a line from the anterior superior iliac spine to the center of the patella and a line from the patella center to the tibial tuberosity, reflecting frontal-plane lower-limb alignment [34], were measured using PSM. Both methods are validated and have demonstrated good reliability and validity for clinical posture assessment [35].

#### 2.2.2. Dynamic Parameters

Dynamic spinopelvic parameters were recorded during treadmill walking at speeds of 1, 2, 4, and 5 km/h using the same rasterstereographic system. The measured parameters included the same spinopelvic variables as in the static assessment. Dynamic measurements were continuously collected throughout the gait cycle and averaged across multiple steps during the steady-state phase at each walking speed [36].

### 2.3. Procedures

#### 2.3.1. Participant Preparation

Participants were instructed to avoid strenuous physical activity for 24 h before testing to minimize fatigue related postural changes. They wore minimal clothing to expose anatomical landmarks (shorts; females wore gowns secured anteriorly), removed jewelry and footwear, and tied long hair to ensure clear visibility.

#### 2.3.2. CVA and Q-Angle Assessment

CVA and Q-angle were assessed using PostureScreen^®^ Mobile (PSM) in standardized weight-bearing positions. For CVA, participants stood barefoot in a natural upright stance with arms relaxed at the sides. Standardized photographs were captured from anterior, posterior, and lateral views. Anatomical landmarks, including the tragus and C7 vertebra, were manually identified on the software, which then automatically calculated linear displacements and angular deviations to quantify head and cervical alignment relative to the trunk.

Q-angle was assessed bilaterally from the same anterior photograph used for CVA. Participants stood barefoot with knees fully extended, patellae facing forward, feet shoulder width apart, and arms relaxed at the sides. Anatomical landmarks, including the anterior superior iliac spine, center of the patella, and tibial tuberosity, were digitized on both limbs. PSM automatically computed Q-angle values for the right and left sides, and the mean of these bilateral measurements was used for statistical analysis to represent overall frontal plane lower-limb alignment under weight-bearing conditions. Static standing Q-angle was measured as a widely used, clinically feasible index of lower-limb alignment. Measuring Q-angle in the weight-bearing standing posture has been shown to better reflect functional lower-limb alignment and allows integration with global spinopelvic parameters in routine posture assessment [37,38].

#### 2.3.3. Static Spinopelvic Assessment

**Figure 2:**
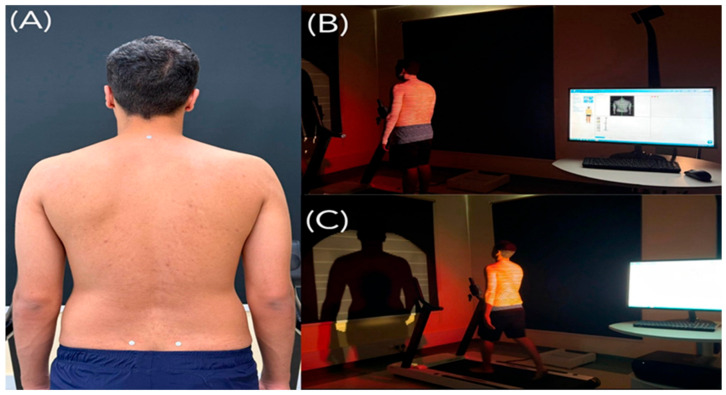
Illustration of marker placement and rasterstereographic measurements using the DIERS formetric system: (**A**) Placement of reflective markers on the posterior superior iliac spines (PSISs) and the vertebra prominens (C7). (**B**) Static posture assessment captured by the DIERS system. (**C**) Dynamic measurement during treadmill based gait analysis using the same device.

Participants stood in a neutral upright posture, with arms relaxed at the sides, knees extended, and gaze directed forward, following recommended rasterstereographic assessment procedures [32]. Rasterstereographic scans were performed with the camera positioned at a fixed distance of 2.0 m from the participant’s back, as recommended by the manufacturer [32]. Participants were positioned with their feet between the marked arrows on the DIERS Formetric 4Dmotion^®^ treadmill, and proper placement was verified by measuring the distance from the camera to the marked point using a measuring tape. To enhance anatomical landmark detection and reconstruction accuracy, reflective markers were placed over the spinous process of C7 and on the right and left posterior superior iliac spines (PSISs), consistent with established rasterstereography protocols [36,39] (Figure 2). For static assessment, participants maintained the neutral standing posture for approximately 5 s while the system recorded at 50 frames per second. All frames acquired during this interval (=250 frames) were automatically averaged by DIERS software (v3.17.0.27), to generate a single static three-dimensional reconstruction of the trunk and pelvis. If any visible movement or postural adjustment occurred during acquisition, the measurement was repeated and the trial without artifact was used for analysis. Spinopelvic and spinal parameters were calculated based on the resulting three-dimensional reconstruction.

#### 2.3.4. Dynamic Spinopelvic Assessment

To accurately capture dynamic spinopelvic behavior, participants were first instructed to stand upright in a neutral posture, with arms relaxed at their sides, on the DIERS Formetric 4Dmotion^®^ treadmill. They were positioned approximately 2.0 m from the system’s camera, and proper placement was verified by measuring the distance from the camera to the marked reference point using a measuring tape to ensure consistent alignment and optimal image acquisition [32]. For each walking speed (1, 2, 4, and 5 km/h), participants walked until a visually confirmed steady-state gait pattern was established (approximately 20–30 s). Dynamic data were then recorded continuously over a 30–40 s interval, capturing trunk and pelvic kinematics at 50 frames per second. This interval included approximately 25–40 consecutive gait cycles, depending on speed. Spinopelvic parameters were averaged across all gait cycles to produce a single representative dynamic value for each outcome. Trials showing irregular gait, loss of balance, or stepping adjustments were repeated to ensure data quality.

### 2.4. Sample Size Determination

An a priori power analysis was conducted using G*Power 3.1 for a multiple linear regression model with 10 static predictors, assuming a moderate effect size (f^2^ = 0.15) in line with Cohen’s conventions, a two-tailed α = 0.05, and power (1 − β) = 0.80. The required minimum sample size was 89 participants. To ensure adequate model stability and allow for potential outliers, we recruited 100 healthy young adults, which exceeded the minimum required threshold.

### 2.5. Data Analysis

All statistical analyses were performed using IBM SPSS Statistics (Version 29.0; IBM Corp., Armonk, NY, USA). Descriptive statistics (mean ± SD) were calculated for demographic variables, static global alignment parameters, and dynamic spinopelvic outcomes.

Separate multiple linear regression (MLR) models were constructed for each walking speed (1, 2, 4, and 5 km/h) to examine the predictive value of static global alignment parameters for dynamic spinopelvic outcomes. Predictor variables included sagittal imbalance (anterior–posterior trunk shift), coronal imbalance (lateral deviation), pelvic rotation, pelvic torsion, pelvic obliquity, vertebral rotation, thoracic kyphosis angle, lumbar lordosis angle, pelvic tilt, craniovertebral angle (CVA), and Q-angle. Dependent variables were dynamic sagittal imbalance, coronal imbalance, pelvic rotation, pelvic torsion, pelvic obliquity, vertebral rotation, thoracic kyphosis, lumbar lordosis, and pelvic tilt.

Predictors were checked for multicollinearity using variance inflation factors (VIFs), with values below 5 indicating acceptable collinearity. Model assumptions, including normality of residuals (Shapiro–Wilk test), homoscedasticity (Breusch–Pagan test), and independence of errors (Durbin–Watson statistic), were evaluated for each model. Standardized beta coefficients (β), standard errors (SE), 95% confidence intervals (CI), and *p*-values were reported for all predictors, regardless of statistical significance, alongside model fit indices (adjusted R^2^ and F-statistics).

All tests were two-tailed, and a significance level of *p* < 0.05 was adopted. Model performance and predictive accuracy were further examined through adjusted R^2^ values and residual diagnostic plots.

## 3. Results

This section presents the results of the predictive analysis evaluating how static spinopelvic alignment parameters, measured at rest (speed = 0 km/h), predict dynamic spinopelvic behavior across different walking speeds (1, 2, 4, and 5 km/h). Unless otherwise stated, all angular measures are reported in degrees (°), linear positional measures in millimeters (mm), and the total sample size was *n* = 100.

### 3.1. Descriptive Statistics

Table 2 summarizes descriptive statistics for all static predictors (measured at rest) and pooled dynamic outcomes. Static parameters showed moderate variability, particularly kyphotic and lordotic angles, while CVA and Q-angle displayed substantial inter-individual differences. Dynamic outcome values presented here are pooled across all walking speeds.

### 3.2. Model Diagnostics and Collinearity

**Figure 3:**
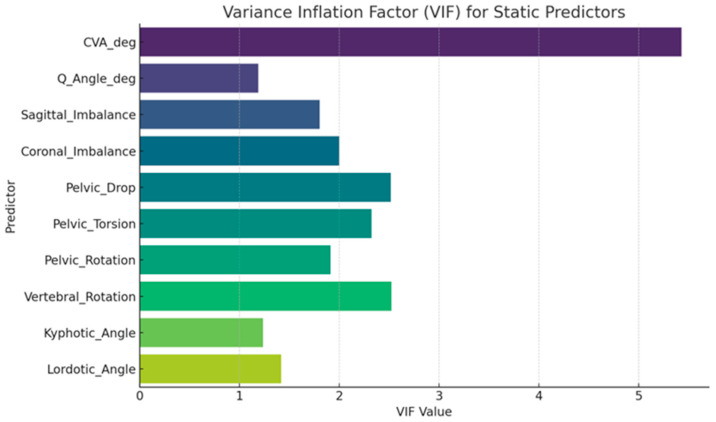
VIF values for each static predictor. The highest VIF is observed for CVA_deg, indicating some multicollinearity, but all values remain below the critical threshold of 10, suggesting acceptable levels of redundancy among predictors.

Variance Inflation Factor (VIF) values were computed for each static predictor to evaluate multicollinearity. All predictors were standardized before calculation. A VIF > 10 typically indicates problematic multicollinearity (Figure 3).

### 3.3. Speed-Stratified Prediction (Multiple Linear Regression)

Separate multiple linear regression (MLR) models were constructed for each walking speed (1, 2, 4, and 5 km/h) to evaluate the predictive power of static alignment parameters for dynamic spinopelvic outcomes. Predictor variables included CVA, Q-angle, sagittal and coronal imbalance, pelvic rotation, pelvic torsion, pelvic obliquity, vertebral rotation, kyphotic angle, lordotic angle, and pelvic tilt.

**Figure 4:**
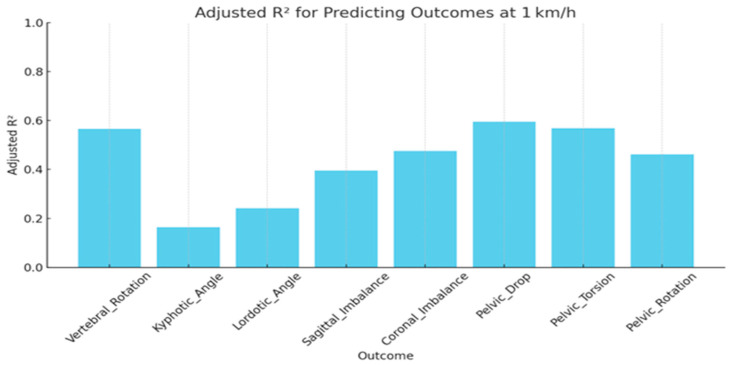
Predictive accuracy of Dynamic Spinopelvic Outcomes (R^2^ values) at 1 km/h.

At 1 km/h (slow, feedback-dominant gait), vertebral rotation demonstrated the highest predictive power, with an adjusted R^2^ of 0.566, indicating that over 56% of its variance was explained by static predictors including CVA and Q-angle. Coronal and sagittal imbalance also showed moderate predictive strength (adjusted R^2^ = 0.476 and 0.396, respectively), whereas lordotic and kyphotic angles were less well predicted (adjusted R^2^ = 0.242 and 0.164) (Table 3 and Figure 4).

**Figure 5:**
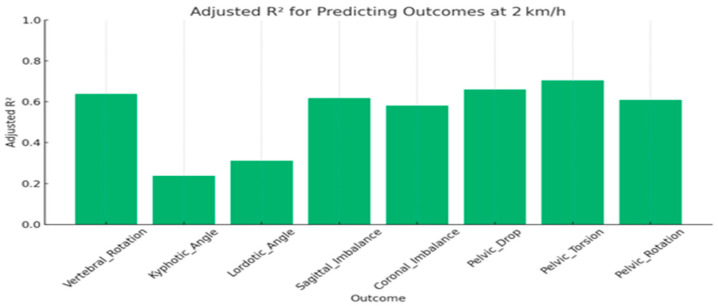
Predictive accuracy Dynamic Spinopelvic Outcomes (R^2^ values) at 2 km/h.

At 2 km/h, vertebral rotation, sagittal imbalance, and coronal imbalance remained strongly predicted by static parameters, with adjusted R^2^ values exceeding 0.58, while lordotic and kyphotic angles accounted for only 24–31% of their variance. Additional pelvic measures, including pelvic drop, torsion, and rotation, demonstrated robust predictive performance (adjusted R^2^ = 0.61–0.71) (Table 4 and Figure 5).

**Figure 6:**
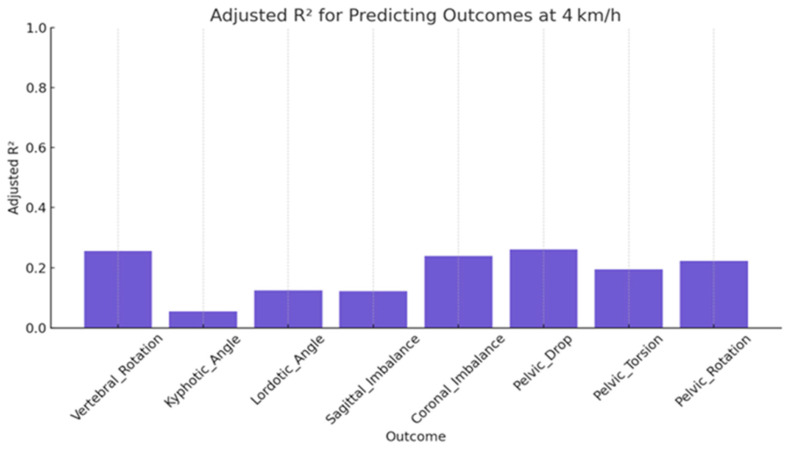
Predictive accuracy of Dynamic Spinopelvic Outcomes (R^2^ values) at 4 km/h.

For 4 km/h, all models exhibited reduced explanatory power. Only vertebral rotation and coronal imbalance reached modest predictive levels (adjusted R^2^~0.25), while the remaining outcomes, including kyphotic angle, showed minimal variance explained, with the kyphotic angle model failing to reach significance (*p* = 0.118) (Table 5 and Figure 6).

**Figure 7:**
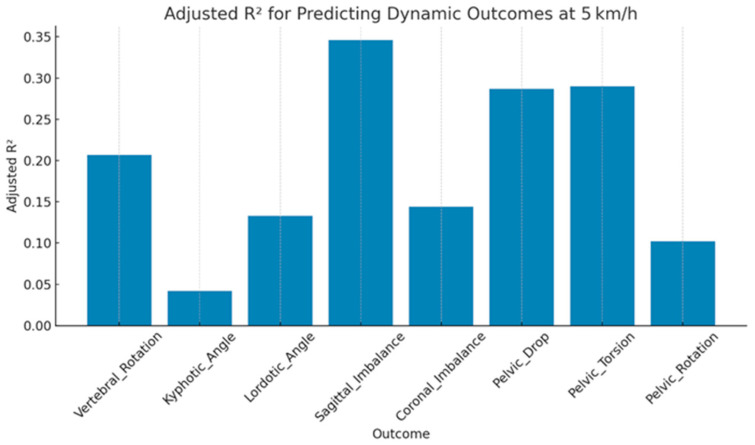
Predictive accuracy of Dynamic Spinopelvic Outcomes (R^2^ values) at 5 km/h.

At 5 km/h, Among the outcomes evaluated at 5 km/h, sagittal imbalance emerged as the most robustly predicted variable, with an adjusted R^2^ of 0.35, indicating a moderate proportion of variance explained by the static alignment predictors. In contrast, kyphotic angle demonstrated the weakest predictive performance, with a non-significant model (*p* = 0.166) and minimal explanatory value. The root mean square error (RMSE) values ranged from 0.77 to 0.93 standardized units, reflecting moderate predictive error magnitudes and suggesting reduced model accuracy relative to lower walking speeds (e.g., 1–2 km/h). Although models for coronal imbalance and lordotic angle reached statistical significance (*p* < 0.01), their relatively low adjusted R^2^ values (0.14 and 0.13, respectively) highlight limited predictive utility, potentially reflecting weak or nonlinear associations with static postural parameters (Table 6 and Figure 7).

**Figure 8:**
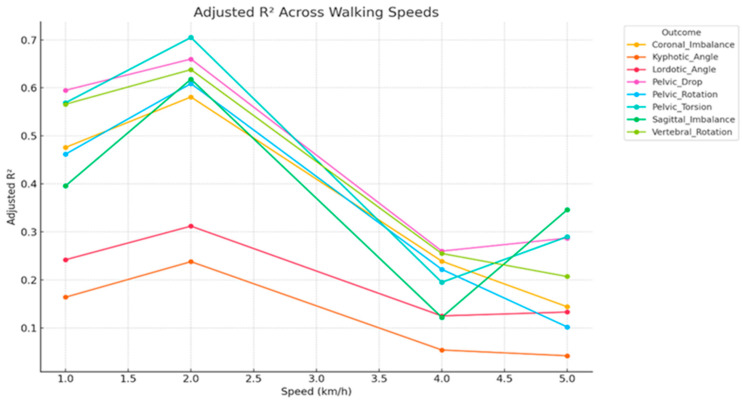
Adjusted R^2^ values for multiple linear regression models predicting dynamic spinopelvic outcomes across walking speeds (1, 2, 4, and 5 km/h). Each line represents a unique outcome variable predicted from static postural parameters. Higher Adjusted R^2^ indicates greater predictive accuracy and model explanatory power.

Figure 8 illustrates how the predictive strength of static spinopelvic alignment parameters varies with walking speed across eight dynamic outcome variables. Notably, predictive accuracy peaked at 2 km/h for all outcomes, with Pelvic Torsion (Adj. R^2^ = 0.71) and Pelvic Drop (Adj. R^2^ ≈ 0.66) exhibiting the highest explained variance. These findings suggest that moderate-speed walking may elicit kinematic patterns most strongly coupled to static alignment, possibly due to optimal neuromuscular stability at this pace. Conversely, predictive performance declined substantially at 4 and 5 km/h, particularly for Sagittal Imbalance (decreasing from 0.61 to 0.29) and Pelvic Rotation (from 0.60 to 0.10), implying that at higher speeds, dynamic compensations and variability in motor control override static structural influences, thereby reducing the models’ explanatory power. Additionally, Kyphotic and Lordotic Angles consistently demonstrated the weakest model performance across all speeds (Adj. R^2^ < 0.31), indicating that these spinal curvatures may not be reliably predicted from external static posture metrics and might require more sensitive biomechanical assessments (e.g., radiographic imaging) for accurate estimation. The pronounced decline in Adjusted R^2^ beyond 2 km/h across most outcomes underscores a limitation of using static measures for dynamic prediction at faster gaits, reinforcing the need for dynamic assessments or advanced modeling approaches (e.g., nonlinear models, regularized regression) to better capture complex movement behavior.

### 3.4. Cross-Validated Performance

**Figure 9:**
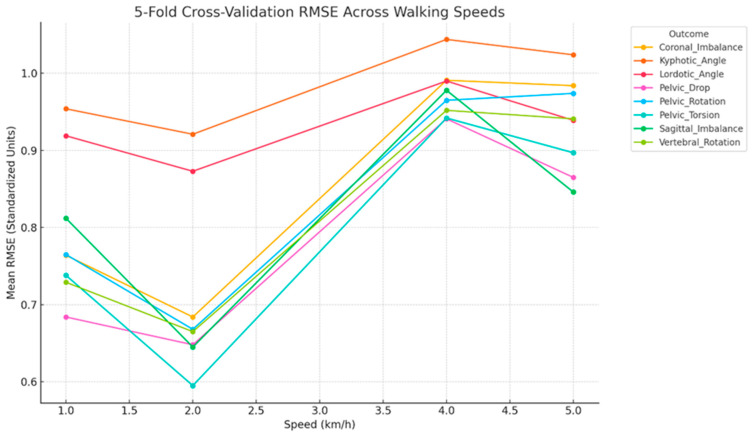
The cross-validation error analysis summarizes the predictive performance (R^2^ and RMSE) of static alignment parameters across walking speeds (1, 2, 4, and 5 km/h).

The cross-validation error analysis summarizes model predictive performance (R^2^) for each dynamic outcome across walking speeds of 1, 2, 4, and 5 km/h, providing insight into how effectively static postural features predict dynamic motion under varying gait demands (Figure 9). The analysis offers a robust estimate of the generalizability of linear regression models linking static alignment parameters to dynamic spinopelvic outcomes. Mean RMSE values across these speeds reveal distinct trends in prediction accuracy relative to locomotor intensity. Nearly all outcomes demonstrated their lowest cross-validated RMSE at 2 km/h, identifying this speed as the point of maximum biomechanical predictability. This suggests a biomechanical “sweet spot” in which gait stability is optimized and neuromuscular compensations are minimal, allowing static alignment parameters to most closely map onto dynamic behavior. Pelvic Torsion and Pelvic Drop exhibited the strongest predictive coupling with RMSE values between 0.60 and 0.68, while Sagittal Imbalance also showed relatively low error, supporting its potential as a clinically relevant indicator for posture-based interventions. In contrast, prediction error increased substantially at higher speeds (4–5 km/h), particularly for Kyphotic Angle (RMSE ≈ 0.95–1.05), reaffirming its weak and unstable relationship with static postural metrics. Similarly, Coronal Imbalance and Pelvic Rotation demonstrated sharp rises in error, indicating that as gait speed increases, compensatory or dynamic control mechanisms may override static structural influences. Collectively, these findings highlight that static postural alignment parameters provide stronger predictive value at moderate walking speeds, while their explanatory power diminishes when motor variability and neuromuscular adaptation dominate at higher locomotion intensities.

### 3.5. Summary of Key Predictors Across Outcomes and Walking Speeds

Across all dynamic outcomes and walking speeds, CVA, Q-angle, and pelvic rotational measures consistently emerged as the strongest predictors. At slower speeds (1–2 km/h), several dynamic parameters demonstrated significant associations with static predictors particularly CVA and pelvic rotation while predictive strength declined markedly at higher speeds (4–5 km/h).

A complete breakdown of the multiple regression results, including standardized β coefficients, 95% confidence intervals, and *p*-values for all predictors across outcomes and speeds, is provided in the Supplementary Materials (Supplementary Table S1).

## 4. Discussion

This study investigated how static postural alignment parameters predict dynamic spinopelvic behavior across different walking speeds in healthy young adults. The findings demonstrate a clear velocity-dependent relationship between structural alignment and dynamic control. At slow to moderate speeds (1–2 km/h), static posture variables, particularly craniovertebral angle (CVA), pelvic torsion, pelvic drop, and sagittal imbalance, explained a meaningful proportion of the variance in dynamic spinopelvic outcomes. In contrast, predictive capacity decreased markedly beyond 2 km/h, indicating that static posture alone becomes insufficient to anticipate gait behavior once locomotion is governed by neuromuscular strategies. These insights refine current understanding of the posture–movement continuum and offer clinically actionable implications.

Furthermore, our results are consistent with longitudinal and repeated-measure studies indicating that static postural alignment can influence dynamic gait adaptations over time and under different functional conditions. For example, increases in gait speed, load carriage, or walking on inclines/declines have been shown to amplify trunk–pelvis coordination demands and pelvic compensation, reflecting dynamic adjustments in spinopelvic control [21,22,23].

### 4.1. Biomechanical Role of Key Static Predictors

Several static parameters emerged as central drivers of dynamic spinopelvic control. Smaller CVA values reflecting forward head posture were associated with anterior displacement of the body’s center of mass, altering trunk-pelvis coupling and increasing compensatory demands along the posterior kinetic chain. These compensations often manifest as lumbar extension and pelvic counterrotation, consistent with prior evidence linking cervical alignment to global balance and gait adaptations [12,21,40,41].

Pelvic torsion and pelvic drop were also highly predictive at lower velocities, underscoring the pelvis as the primary mechanical transmission hub between the trunk and lower limbs [42]. Deviations in pelvic symmetry and rotation influence energy transfer, frontal plane stabilization, and lumbosacral rhythm, thereby shaping how movement unfolds dynamically. This centrality of pelvic alignment is consistent with reports showing that pelvic asymmetries amplify trunk sway, modify stride parameters, and disrupt intersegmental coordination [42,43]. Notably, spinal curvature indices thoracic kyphosis and lumbar lordosis demonstrated weaker predictive strength, likely reflecting their compensatory rather than primary role in regulating locomotor mechanics [44].

The Q-angle, hypothesized to contribute to altered frontal plane mechanics [3,45], showed limited and inconsistent associations. This suggests that lower limb alignment indicators possess secondary influence, likely mediated indirectly through hip–pelvic mechanics rather than exerting direct control over spinopelvic coordination. Collectively, these findings reinforce the idea that postural drivers of dynamic stability originate proximally at the cervical and pelvic levels rather than distally in the lower limb [45].

### 4.2. Transition to Neuromuscular Control at Higher Speeds

The gradual erosion of predictive accuracy at higher speeds reflects a fundamental shift in gait regulation. At slower velocities, locomotion is predominantly feedback-driven, relying on continuous sensory integration from visual, vestibular, and proprioceptive sources to maintain equilibrium [46]. Under these conditions, structural misalignments are expressed directly in dynamic movement, as corrective responses are executed in real time. Consequently, static posture serves as a meaningful proxy for explaining variability in spinopelvic behavior [47].

Beyond ~2 km/h, locomotion becomes increasingly feedforward-dominant, engaging anticipatory strategies, central pattern generator activity, and momentum-based stabilization [48]. During fast gait, joint moments, ground reaction forces, and segmental kinetics increase disproportionately, reducing the influence of initial alignment [49]. The nervous system prestructures movement patterns in advance, leveraging neuromotor programs rather than real-time corrections. As a result, static predictors such as CVA or pelvic geometry that effectively modeled 1–2 km/h performance no longer captured dynamic behavior at 4–5 km/h [50,51]. This shift explains the consistent drop in adjusted R^2^ values at higher velocities and underscores the limits of posture-centric assessment when neuromotor mechanisms dominate.

### 4.3. Neural Integration: Cervical Inputs and Pelvic Coupling

The neurophysiological mechanisms underpinning the observed patterns offer additional insight. The upper cervical spine is densely populated with muscle spindles and mechanoreceptors crucial for proprioception and reflexive head-trunk coordination [52]. Alterations in cervical alignment such as forward head posture distort afferent signals, impair vestibulo-ocular and cervicocollic reflexes, and delay anticipatory stabilization responses [53]. This disruption may degrade trunk–pelvis communication, explaining why reduced CVA was associated with changes in pelvic rotation and torsion in our slow-velocity models.

Similar neurophysiological mechanisms have been documented in individuals with chronic neck dysfunction, idiopathic scoliosis, or proprioceptive deficits, who demonstrate greater sway, altered muscle activation timing, and instability during dynamic task [29,54]. Importantly, the alignment prediction weakens when locomotion is driven predominantly by automatic spinal circuitry, as seen in high-speed movement. Under such conditions, the nervous system prioritizes forward propulsion and momentum management rather than sensory correction, diminishing the explanatory power of static markers.

### 4.4. Clinical Implications and Functional Applications

The findings of this study have direct relevance for clinical practice, fall prevention, and athletic rehabilitation [25,55,56]. CVA and Q-angle can serve as practical screening markers for identifying individuals whose static postural configurations may predispose them to instability, particularly in early-stage rehabilitation, older adults, or populations at risk of falls [57,58]. Their predictive value is most pronounced during slow, posture-dominated gait (1–2 km/h), where static alignment parameters significantly influence spinopelvic dynamics. Assessment of gait at these lower speeds appears more sensitive for detecting structure-based deviations, enabling clinicians to target corrective interventions before faster walking speeds shift locomotor control toward neuromuscular strategies that diminish the influence of static posture [59,60]. The present findings therefore suggest that static-alignment–driven deviations have the greatest impact during low velocity, feedback dominant locomotion.

Accordingly, early rehabilitation should emphasize interventions that target structural alignment and sensorimotor recalibration. These may include cervical proprioceptive retraining to improve cervicoocular and cervicovestibular reflex integration [61], pelvic stability protocols that focus on hip abductor and rotator strengthening with frontal plane motor control, and segmental postural correction strategies such as spinal extension bias, scapular retraction drills, or pelvic leveling. These posture-oriented interventions may enhance balance and spinopelvic control during controlled, feedback-driven locomotion, allowing clinicians to address alignment-related deficiencies before neuromuscular adaptations take precedence.

In contrast, for higher gait speeds (≥4 km/h), where static predictors lose explanatory strength, rehabilitation should progressively shift toward enhancing neuromuscular feedforward control. This transition may include agility and dual-task training, anticipatory balance strategies, perturbation-based treadmill programs, and progressive sport-specific movements. In sports and high-performance rehabilitation contexts, where walking or running speed substantially increases reliance on feedforward mechanisms, posture-focused interventions alone may be insufficient. Instead, they should be integrated with neuromuscular and proprioceptive training to optimize anticipatory control, improve dynamic stability, and complement static alignment correction under high-speed conditions.

Overall, this gait-speed–dependent progression provides clinicians with a structured and evidence-based framework for aligning postural correction with neuromotor demands across different stages of rehabilitation, thereby enhancing both balance control and functional stability.

### 4.5. Limitations and Future Directions

Although the current study utilized experimentally acquired cross-sectional data from healthy young adults, the predictive models developed here still require validation in broader and clinically diverse populations. The sample consisted exclusively of healthy young adults aged 18–25 years, which helped reduce confounding from age-related degenerative changes, osteopenia/osteoporosis, or chronic musculoskeletal pain, and provided a homogeneous normative reference for the posture–gait relationship. However, the present findings cannot be directly extrapolated to middle-aged adults (30–60 years) or older adults (>65 years), who typically exhibit age-related postural adaptations, reduced gait reserve, and greater fall risk.

The use of convenience sampling from a young, physically active university population may have restricted variability and limited generalizability to older or clinical cohorts with broader alignment deviations. The restricted age range also excludes populations in which posture-related compensations and neurodegenerative gait patterns are more prominent. Additionally, rasterstereography captures surface-based representations of spinal geometry, which may underestimate deeper vertebral rotation compared with biplanar or radiographic methods. Furthermore, the predictive analyses assumed linear relationships, whereas neuromotor regulation of fast gait is likely nonlinear and adaptive. These dynamics could be better modeled using advanced analytical frameworks such as regularized regression, nonlinear kernels, or deep learning approaches.

Future longitudinal work should extend this approach to clinical and aging cohorts, including individuals with postural deformities, musculoskeletal pain, or balance impairments groups in which functional instability and falls are more prevalent. Future studies should also incorporate kinetic measures (e.g., ground reaction forces, joint moments) and electromyographic synergy analyses to better characterize neuromuscular coordination. Incorporating dual-task gait paradigms or mechanical perturbation tests could further elucidate compensatory mechanisms under cognitive or external load.

Finally, longitudinal investigations are warranted to determine whether poor craniovertebral angle (CVA), pelvic asymmetry, or abnormal Q-angle profiles predict injury, musculoskeletal pain, or fall risk over time, and to examine whether similar velocity dependent patterns of spinopelvic control are preserved or altered across aging and clinical populations.

## 5. Conclusions

This study demonstrates a clear velocity-dependent relationship between static postural alignment and dynamic spinopelvic control. At slow walking speeds (1–2 km/h), static parameters, particularly craniovertebral angle (CVA), pelvic torsion, pelvic drop, and sagittal imbalance, moderately predicted dynamic outcomes, reflecting the dominance of feedback-driven postural regulation. Q-angle showed limited and inconsistent predictive value, suggesting a secondary role in influencing spinopelvic behavior.

As gait speed increased, the predictive strength of static alignment measures declined substantially, highlighting a progressive transition toward feedforward neuromuscular strategies and anticipatory control mechanisms that static metrics alone cannot capture.

These findings underscore the importance of comprehensive gait assessment across multiple speeds and support the integration of postural screening with dynamic neuromuscular evaluation in clinical practice. Rehabilitation strategies that address both structural alignment and motor control may enhance functional stability and reduce the risk of musculoskeletal dysfunction during locomotion.

## Figures and Tables

**Table 1:** Definitions and Interpretations of Rasterstereographic Spinopelvic Parameters.

Parameters	Definitions and Interpretations
(A) Sagittal Imbalance	The vertical difference in height between the vertebra prominens (VP) and the midpoint between the left and right posterior superior iliac spines (dimple middle, DM) in the sagittal plane.
(B) Coronal Imbalance	The lateral deviation of the VP from the DM. A positive value indicates a shift in the VP to the right, while a negative value indicates a shift to the left.
(C) Kyphotic Angle	The maximum kyphotic angle measured between the surface tangents at the upper inflection point (near the VP) and the thoracolumbar inflection point.
(D) Lordotic Angle	The maximum lordotic angle measured between the surface tangents at the thoracolumbar inflection point and the lumbosacral inflection point.
(E) Vertebral Rotation	The root mean square (RMS) of the horizontal components of the surface normals along the spinal symmetry line.
(F) Pelvic Obliquity	The vertical difference in height between the left and right lumbar dimples.
(G) Pelvic Torsion	The torsional rotation difference between the surface normals at the two lumbar dimples.
(H) Pelvic Rotation	The horizontal rotation of the right dimple (DR) relative to the left dimple (DL), based on a frontal plane passing through the DL.
(I) Pelvic Tilt (drop)	The difference in height of the lumbar dimples based on horizontal plane. DR is higher than DL if the angle is positive.

**Table 2:** Summary statistics for static predictors and dynamic outcomes.

Variable	Mean	SD	Min	Max
Age (years)	21.6	2.4	18	27
Height (cm)	168.4	7.9	154	189
Weight (kg)	65.3	10.8	46	94
BMI (kg/m^2^)	22.9	3.0	18.3	30.7
Gender, *n* (%)	Female: 58 (58%) Male: 42 (42%)	-	-	-
Self-reported physical activity (IPAQ categories)		-	-	-
1. Low	15 (15%)	-	-	-
2. Moderate	67 (67%)	-	-	-
3. High	18 (18%)	-	-	-
Occupation				
1. Undergraduate students	90 (90%)	-	-	-
2. Graduate students	4 (4%)	-	-	-
3. University staff	6 (6%)	-	-	-
CVA (°)	51.23	6.50	40.66	61.38
Q-Angle (°)	14.83	3.34	7.09	23.24
Sagittal Imbalance_0_ (mm)	3.93	0.97	1.68	6.02
Coronal Imbalance_0_ (mm)	5.52	1.16	2.95	8.27
Pelvic Drop_0_ (°)	3.25	1.03	1.16	5.37
Pelvic Torsion_0_ (°)	3.39	0.88	1.83	5.53
Pelvic Rotation_0_ (°)	4.29	1.08	1.76	6.81
Vertebral Rotation_0_ (°)	5.27	1.15	2.60	7.93
Kyphotic Angle_0_ (°)	56.73	2.80	50.97	64.10
Lordotic Angle_0_ (°)	42.91	2.72	36.46	49.10
Sagittal Imbalance (mm)	9.18	5.09	1.80	19.96
Coronal Imbalance (mm)	13.23	7.31	3.16	27.35
Pelvic Drop (°)	9.56	6.03	1.24	21.80
Pelvic Torsion (°)	8.61	5.03	1.90	19.99
Pelvic Rotation (°)	10.56	5.89	2.02	23.82
Vertebral Rotation (°)	11.48	5.95	2.82	24.86
Kyphotic Angle (°)	58.45	3.92	50.51	70.12
Lordotic Angle (°)	43.63	3.34	35.33	54.05

**Table 3:** Predictive Accuracy of Dynamic Spinopelvic Outcomes at 1 km/h.

Outcome	Adjusted R^2^	MAE	RMSE	F-Stat	*p*-Value
Vertebral Rotation	0.57	0.50	0.63	15.32	<0.001
Coronal Imbalance	0.48	0.56	0.69	11.01	<0.001
Sagittal Imbalance	0.40	0.58	0.74	8.22	<0.001
Lordotic Angle	0.24	0.67	0.83	4.52	<0.001
Kyphotic Angle	0.16	0.69	0.87	3.17	0.002

**Table 4:** Predictive Accuracy of Dynamic Spinopelvic Outcomes at 2 km/h.

Outcome	Adjusted RÂ^2^	MAE	RMSE	F-Stat	*p*-Value
Vertebral_Rotation	0.64	0.46	0.57	20.35	<0.001
Kyphotic_Angle	0.24	0.69	0.83	4.44	<0.001
Lordotic_Angle	0.31	0.62	0.79	5.98	<0.001
Sagittal_Imbalance	0.62	0.46	0.59	18.79	<0.001
Coronal_Imbalance	0.58	0.50	0.62	16.24	<0.001
Pelvic_Drop	0.66	0.46	0.56	22.35	<0.001
Pelvic_Torsion	0.71	0.41	0.52	27.29	<0.001
Pelvic_Rotation	0.61	0.48	0.60	18.12	<0.001

**Table 5:** Predictive Accuracy of Dynamic Spinopelvic Outcomes at 4 km/h.

Outcome	Adjusted RÂ^2^	MAE	RMSE	F-Stat	*p*-Value
Vertebral_Rotation	0.26	0.67	0.82	4.77	<0.001
Kyphotic_Angle	0.05	0.75	0.93	1.63	0.118
Lordotic_Angle	0.13	0.73	0.89	2.56	0.011
Sagittal_Imbalance	0.12	0.72	0.89	2.53	0.012
Coronal_Imbalance	0.24	0.67	0.83	4.45	<0.001
Pelvic_Drop	0.26	0.64	0.82	4.87	<0.001
Pelvic_Torsion	0.05	0.70	0.86	3.66	<0.001
Pelvic_Rotation	0.13	0.67	0.84	4.15	<0.001

**Table 6:** Predictive Accuracy of Dynamic Spinopelvic Outcomes at 5 km/h.

Outcome	Adjusted RÂ^2^	MAE	RMSE	F-Stat	*p*-Value
Vertebral_Rotation	0.21	0.70	0.85	3.88	<0.001
Kyphotic_Angle	0.04	0.75	0.93	1.48	0.166
Lordotic_Angle	0.13	0.71	0.89	2.69	0.008
Sagittal_Imbalance	0.35	0.60	0.77	6.81	<0.001
Coronal_Imbalance	0.14	0.72	0.88	2.86	0.005
Pelvic_Drop	0.29	0.65	0.81	5.43	<0.001
Pelvic_Torsion	0.29	0.63	0.80	5.49	<0.001
Pelvic_Rotation	0.10	0.71	0.90	2.25	0.025

## Data Availability

All data generated or analyzed during this study are included in this published article and its Supplementary Materials.

## Supplementary Materials

The following supporting information can be downloaded at: https://www.mdpi.com/article/10.3390/jcm15010073/s1, A summary of the key predictors of dynamic spinopelvic outcomes across walking speeds can be found in the Supplementary Materials (Supplementary Table S1).

## Abbreviations

The following abbreviations are used in this manuscript:

CVA	Craniovertebral Angle
FHP	Forward head posture
Q-angle	Quadriceps angle
