# Supplementary material for: Modeling the Posture–Movement Continuum: Predictive Mapping of Spinopelvic Control Across Gait Speeds
Source: J Clin Med. 2025 Dec 22;15(1):73. doi: 10.3390/jcm15010073 (PMC12787025; doi:10.3390/jcm15010073)
Supplement: Supplementary file 1 [file jcm-15-00073-s001.zip › jcm-4007394-supplementary.pdf]

Supplementary Tables

Supplementary Table S1. Summary of Key Predictors of Dynamic Spinopelvic Outcomes Across Walking Speeds.

Speed (km/h)	Outcome	Predictor	Standardized Î²	95% CI	p-value
1	Vertebral_Rotation	Intercept	0	[-0.132, 0.132]	1
1	Vertebral_Rotation	CVA_deg	-0.682	[-0.96, -0.404]	0
1	Vertebral_Rotation	Q_Angle_deg	0.029	[-0.114, 0.173]	0.6875
1	Vertebral_Rotation	Pelvic_Torsion	-0.129	[-0.338, 0.079]	0.2213
1	Vertebral_Rotation	Pelvic_Rotation	0.052	[-0.136, 0.24]	0.5855
1	Vertebral_Rotation	Pelvic_Drop	0.243	[0.032, 0.454]	0.0243
1	Vertebral_Rotation	Kyphotic_Angle	-0.01	[-0.161, 0.141]	0.8936
1	Vertebral_Rotation	Lordotic_Angle	-0.039	[-0.197, 0.12]	0.6276
1	Vertebral_Rotation	Sagittal_Imbalance	-0.008	[-0.185, 0.17]	0.931
1	Vertebral_Rotation	Coronal_Imbalance	-0.028	[-0.219, 0.162]	0.7687
1	Kyphotic_Angle	Intercept	0	[-0.183, 0.183]	1
1	Kyphotic_Angle	CVA_deg	-0.015	[-0.448, 0.418]	0.9455
1	Kyphotic_Angle	Q_Angle_deg	-0.122	[-0.319, 0.076]	0.2232
1	Kyphotic_Angle	Pelvic_Torsion	0.163	[-0.127, 0.452]	0.2675
1	Kyphotic_Angle	Pelvic_Rotation	-0.053	[-0.314, 0.208]	0.6861
1	Kyphotic_Angle	Pelvic_Drop	0.187	[-0.112, 0.485]	0.2173
1	Kyphotic_Angle	Vertebral_Rotation	-0.02	[-0.31, 0.271]	0.8936
1	Kyphotic_Angle	Lordotic_Angle	0.188	[-0.028, 0.404]	0.0877
1	Kyphotic_Angle	Sagittal_Imbalance	0.122	[-0.123, 0.367]	0.3256
1	Kyphotic_Angle	Coronal_Imbalance	0.066	[-0.198, 0.33]	0.6217
1	Lordotic_Angle	Intercept	0	[-0.174, 0.174]	1
1	Lordotic_Angle	CVA_deg	-0.412	[-0.815, -0.009]	0.0454
1	Lordotic_Angle	Q_Angle_deg	0.079	[-0.11, 0.268]	0.4068
1	Lordotic_Angle	Pelvic_Torsion	-0.079	[-0.356, 0.198]	0.5718
1	Lordotic_Angle	Pelvic_Rotation	0.108	[-0.14, 0.356]	0.3882
1	Lordotic_Angle	Pelvic_Drop	0.274	[-0.007, 0.554]	0.056
1	Lordotic_Angle	Vertebral_Rotation	-0.068	[-0.344, 0.209]	0.6276
1	Lordotic_Angle	Kyphotic_Angle	0.17	[-0.026, 0.367]	0.0877
1	Lordotic_Angle	Sagittal_Imbalance	-0.043	[-0.277, 0.192]	0.7191
1	Lordotic_Angle	Coronal_Imbalance	-0.279	[-0.524, -0.034]	0.0261
1	Sagittal_Imbalance	Intercept	0	[-0.155, 0.155]	1
1	Sagittal_Imbalance	CVA_deg	-0.454	[-0.81, -0.099]	0.0129
1	Sagittal_Imbalance	Q_Angle_deg	0.21	[0.047, 0.374]	0.0123
1	Sagittal_Imbalance	Pelvic_Torsion	-0.04	[-0.287, 0.208]	0.7522
1	Sagittal_Imbalance	Pelvic_Rotation	0.072	[-0.15, 0.293]	0.5206
1	Sagittal_Imbalance	Pelvic_Drop	0.138	[-0.117, 0.392]	0.2849
1	Sagittal_Imbalance	Vertebral_Rotation	-0.011	[-0.258, 0.236]	0.931
1	Sagittal_Imbalance	Kyphotic_Angle	0.088	[-0.089, 0.265]	0.3256

1	Sagittal_Imbalance	Lordotic_Angle	-0.034	[-0.221, 0.153]	0.7191
1	Sagittal_Imbalance	Coronal_Imbalance	-0.016	[-0.24, 0.209]	0.8907
1	Coronal_Imbalance	Intercept	0	[-0.144, 0.144]	1
1	Coronal_Imbalance	CVA_deg	-0.593	[-0.913, -0.273]	0.0004
1	Coronal_Imbalance	Q_Angle_deg	0.019	[-0.138, 0.177]	0.8091
1	Coronal_Imbalance	Pelvic_Torsion	0.172	[-0.056, 0.4]	0.1371
1	Coronal_Imbalance	Pelvic_Rotation	0.053	[-0.154, 0.259]	0.6132
1	Coronal_Imbalance	Pelvic_Drop	0.045	[-0.193, 0.283]	0.7084
1	Coronal_Imbalance	Vertebral_Rotation	-0.034	[-0.264, 0.196]	0.7687
1	Coronal_Imbalance	Kyphotic_Angle	0.041	[-0.124, 0.207]	0.6217
1	Coronal_Imbalance	Lordotic_Angle	-0.193	[-0.362, -0.023]	0.0261
1	Coronal_Imbalance	Sagittal_Imbalance	-0.014	[-0.209, 0.181]	0.8907
1	Pelvic_Drop	Intercept	0	[-0.127, 0.127]	1
1	Pelvic_Drop	CVA_deg	-0.062	[-0.363, 0.239]	0.683
1	Pelvic_Drop	Q_Angle_deg	0.077	[-0.061, 0.215]	0.2685
1	Pelvic_Drop	Pelvic_Torsion	0.239	[0.042, 0.435]	0.018
1	Pelvic_Drop	Pelvic_Rotation	0.152	[-0.027, 0.331]	0.0949
1	Pelvic_Drop	Vertebral_Rotation	0.226	[0.03, 0.423]	0.0243
1	Pelvic_Drop	Kyphotic_Angle	0.09	[-0.054, 0.235]	0.2173
1	Pelvic_Drop	Lordotic_Angle	0.146	[-0.004, 0.296]	0.056
1	Pelvic_Drop	Sagittal_Imbalance	0.092	[-0.078, 0.263]	0.2849
1	Pelvic_Drop	Coronal_Imbalance	0.035	[-0.149, 0.219]	0.7084
1	Pelvic_Torsion	Intercept	0	[-0.131, 0.131]	1
1	Pelvic_Torsion	CVA_deg	-0.544	[-0.833, -0.255]	0.0003
1	Pelvic_Torsion	Q_Angle_deg	0.024	[-0.118, 0.167]	0.7346
1	Pelvic_Torsion	Pelvic_Rotation	0.022	[-0.165, 0.209]	0.8171
1	Pelvic_Torsion	Pelvic_Drop	0.254	[0.045, 0.463]	0.018
1	Pelvic_Torsion	Vertebral_Rotation	-0.128	[-0.335, 0.079]	0.2213
1	Pelvic_Torsion	Kyphotic_Angle	0.084	[-0.065, 0.233]	0.2675
1	Pelvic_Torsion	Lordotic_Angle	-0.045	[-0.203, 0.113]	0.5718
1	Pelvic_Torsion	Sagittal_Imbalance	-0.028	[-0.205, 0.149]	0.7522
1	Pelvic_Torsion	Coronal_Imbalance	0.142	[-0.046, 0.329]	0.1371
1	Pelvic_Rotation	Intercept	0	[-0.146, 0.146]	1
1	Pelvic_Rotation	CVA_deg	-0.395	[-0.733, -0.058]	0.0223
1	Pelvic_Rotation	Q_Angle_deg	-0.077	[-0.236, 0.082]	0.3399
1	Pelvic_Rotation	Pelvic_Torsion	0.027	[-0.207, 0.261]	0.8171
1	Pelvic_Rotation	Pelvic_Drop	0.202	[-0.036, 0.44]	0.0949
1	Pelvic_Rotation	Vertebral_Rotation	0.064	[-0.169, 0.297]	0.5855
1	Pelvic_Rotation	Kyphotic_Angle	-0.034	[-0.202, 0.134]	0.6861
1	Pelvic_Rotation	Lordotic_Angle	0.077	[-0.099, 0.252]	0.3882
1	Pelvic_Rotation	Sagittal_Imbalance	0.064	[-0.133, 0.261]	0.5206
1	Pelvic_Rotation	Coronal_Imbalance	0.054	[-0.158, 0.266]	0.6132
2	Vertebral_Rotation	Intercept	0	[-0.12, 0.12]	1

2	Vertebral_Rotation	CVA_deg	-0.582	[-0.868, -0.296]	0.0001
2	Vertebral_Rotation	Q_Angle_deg	0.024	[-0.11, 0.158]	0.7197
2	Vertebral_Rotation	Pelvic_Torsion	-0.072	[-0.303, 0.16]	0.5403
2	Vertebral_Rotation	Pelvic_Rotation	0.073	[-0.128, 0.274]	0.4749
2	Vertebral_Rotation	Pelvic_Drop	0.148	[-0.066, 0.362]	0.1725
2	Vertebral_Rotation	Kyphotic_Angle	0.022	[-0.122, 0.166]	0.7623
2	Vertebral_Rotation	Lordotic_Angle	0.021	[-0.131, 0.173]	0.7847
2	Vertebral_Rotation	Sagittal_Imbalance	0.065	[-0.139, 0.268]	0.5277
2	Vertebral_Rotation	Coronal_Imbalance	0.051	[-0.143, 0.246]	0.6023
2	Kyphotic_Angle	Intercept	0	[-0.174, 0.174]	1
2	Kyphotic_Angle	CVA_deg	0.039	[-0.411, 0.49]	0.8626
2	Kyphotic_Angle	Q_Angle_deg	-0.126	[-0.319, 0.067]	0.198
2	Kyphotic_Angle	Pelvic_Torsion	0.286	[-0.045, 0.617]	0.0897
2	Kyphotic_Angle	Pelvic_Rotation	-0.019	[-0.311, 0.273]	0.898
2	Kyphotic_Angle	Pelvic_Drop	0.024	[-0.29, 0.337]	0.8801
2	Kyphotic_Angle	Vertebral_Rotation	0.046	[-0.257, 0.35]	0.7623
2	Kyphotic_Angle	Lordotic_Angle	0.27	[0.057, 0.483]	0.0137
2	Kyphotic_Angle	Sagittal_Imbalance	0.205	[-0.087, 0.498]	0.1668
2	Kyphotic_Angle	Coronal_Imbalance	-0.074	[-0.356, 0.208]	0.6038
2	Lordotic_Angle	Intercept	0	[-0.166, 0.166]	1
2	Lordotic_Angle	CVA_deg	-0.182	[-0.609, 0.244]	0.3977
2	Lordotic_Angle	Q_Angle_deg	0.131	[-0.052, 0.313]	0.1595
2	Lordotic_Angle	Pelvic_Torsion	-0.071	[-0.39, 0.249]	0.6602
2	Lordotic_Angle	Pelvic_Rotation	0.144	[-0.133, 0.42]	0.3044
2	Lordotic_Angle	Pelvic_Drop	0.341	[0.052, 0.63]	0.0213
2	Lordotic_Angle	Vertebral_Rotation	0.04	[-0.249, 0.328]	0.7847
2	Lordotic_Angle	Kyphotic_Angle	0.244	[0.051, 0.436]	0.0137
2	Lordotic_Angle	Sagittal_Imbalance	-0.122	[-0.402, 0.158]	0.3877
2	Lordotic_Angle	Coronal_Imbalance	-0.116	[-0.384, 0.151]	0.3893
2	Sagittal_Imbalance	Intercept	0	[-0.123, 0.123]	1
2	Sagittal_Imbalance	CVA_deg	-0.289	[-0.603, 0.024]	0.07
2	Sagittal_Imbalance	Q_Angle_deg	0.224	[0.095, 0.354]	0.0009
2	Sagittal_Imbalance	Pelvic_Torsion	0.146	[-0.09, 0.383]	0.2221
2	Sagittal_Imbalance	Pelvic_Rotation	0.043	[-0.164, 0.249]	0.6828
2	Sagittal_Imbalance	Pelvic_Drop	0.21	[-0.007, 0.428]	0.0582
2	Sagittal_Imbalance	Vertebral_Rotation	0.068	[-0.146, 0.283]	0.5277
2	Sagittal_Imbalance	Kyphotic_Angle	0.103	[-0.044, 0.25]	0.1668
2	Sagittal_Imbalance	Lordotic_Angle	-0.068	[-0.223, 0.088]	0.3877
2	Sagittal_Imbalance	Coronal_Imbalance	0.024	[-0.176, 0.224]	0.8146
2	Coronal_Imbalance	Intercept	0	[-0.129, 0.129]	1
2	Coronal_Imbalance	CVA_deg	-0.486	[-0.804, -0.167]	0.0032
2	Coronal_Imbalance	Q_Angle_deg	0.011	[-0.133, 0.156]	0.8747
2	Coronal_Imbalance	Pelvic_Torsion	0.263	[0.019, 0.506]	0.0346

2	Coronal_Imbalance	Pelvic_Rotation	0.067	[-0.149, 0.283]	0.5391
2	Coronal_Imbalance	Pelvic_Drop	-0.006	[-0.238, 0.227]	0.9604
2	Coronal_Imbalance	Vertebral_Rotation	0.059	[-0.166, 0.284]	0.6023
2	Coronal_Imbalance	Kyphotic_Angle	-0.041	[-0.196, 0.114]	0.6038
2	Coronal_Imbalance	Lordotic_Angle	-0.071	[-0.234, 0.092]	0.3893
2	Coronal_Imbalance	Sagittal_Imbalance	0.026	[-0.193, 0.245]	0.8146
2	Pelvic_Drop	Intercept	0	[-0.116, 0.116]	1
2	Pelvic_Drop	CVA_deg	-0.107	[-0.407, 0.194]	0.4822
2	Pelvic_Drop	Q_Angle_deg	0.004	[-0.126, 0.134]	0.9471
2	Pelvic_Drop	Pelvic_Torsion	0.219	[-0.001, 0.44]	0.0506
2	Pelvic_Drop	Pelvic_Rotation	0.183	[-0.008, 0.375]	0.0607
2	Pelvic_Drop	Vertebral_Rotation	0.139	[-0.062, 0.34]	0.1725
2	Pelvic_Drop	Kyphotic_Angle	0.011	[-0.129, 0.151]	0.8801
2	Pelvic_Drop	Lordotic_Angle	0.169	[0.026, 0.311]	0.0213
2	Pelvic_Drop	Sagittal_Imbalance	0.187	[-0.007, 0.381]	0.0582
2	Pelvic_Drop	Coronal_Imbalance	-0.005	[-0.193, 0.184]	0.9604
2	Pelvic_Torsion	Intercept	0	[-0.108, 0.108]	1
2	Pelvic_Torsion	CVA_deg	-0.456	[-0.72, -0.193]	0.0009
2	Pelvic_Torsion	Q_Angle_deg	-0.007	[-0.128, 0.114]	0.9099
2	Pelvic_Torsion	Pelvic_Rotation	0.013	[-0.168, 0.195]	0.8841
2	Pelvic_Torsion	Pelvic_Drop	0.19	[-0.001, 0.381]	0.0506
2	Pelvic_Torsion	Vertebral_Rotation	-0.058	[-0.247, 0.13]	0.5403
2	Pelvic_Torsion	Kyphotic_Angle	0.111	[-0.018, 0.239]	0.0897
2	Pelvic_Torsion	Lordotic_Angle	-0.03	[-0.167, 0.107]	0.6602
2	Pelvic_Torsion	Sagittal_Imbalance	0.113	[-0.07, 0.295]	0.2221
2	Pelvic_Torsion	Coronal_Imbalance	0.185	[0.014, 0.356]	0.0346
2	Pelvic_Rotation	Intercept	0	[-0.125, 0.125]	1
2	Pelvic_Rotation	CVA_deg	-0.431	[-0.741, -0.121]	0.007
2	Pelvic_Rotation	Q_Angle_deg	-0.033	[-0.172, 0.106]	0.6412
2	Pelvic_Rotation	Pelvic_Torsion	0.018	[-0.223, 0.259]	0.8841
2	Pelvic_Rotation	Pelvic_Drop	0.211	[-0.01, 0.431]	0.0607
2	Pelvic_Rotation	Vertebral_Rotation	0.078	[-0.139, 0.295]	0.4749
2	Pelvic_Rotation	Kyphotic_Angle	-0.01	[-0.16, 0.14]	0.898
2	Pelvic_Rotation	Lordotic_Angle	0.082	[-0.075, 0.239]	0.3044
2	Pelvic_Rotation	Sagittal_Imbalance	0.044	[-0.168, 0.255]	0.6828
2	Pelvic_Rotation	Coronal_Imbalance	0.063	[-0.139, 0.265]	0.5391
4	Vertebral_Rotation	Intercept	0	[-0.172, 0.172]	1
4	Vertebral_Rotation	CVA_deg	-0.583	[-0.856, -0.309]	0.0001
4	Vertebral_Rotation	Q_Angle_deg	0.056	[-0.127, 0.24]	0.5443
4	Vertebral_Rotation	Pelvic_Torsion	0.043	[-0.158, 0.244]	0.6729
4	Vertebral_Rotation	Pelvic_Rotation	0.085	[-0.12, 0.289]	0.4125
4	Vertebral_Rotation	Pelvic_Drop	-0.008	[-0.218, 0.202]	0.938
4	Vertebral_Rotation	Kyphotic_Angle	-0.048	[-0.233, 0.138]	0.6107

4	Vertebral_Rotation	Lordotic_Angle	-0.258	[-0.443, -0.072]	0.007
4	Vertebral_Rotation	Sagittal_Imbalance	-0.011	[-0.204, 0.181]	0.9066
4	Vertebral_Rotation	Coronal_Imbalance	-0.141	[-0.346, 0.064]	0.1748
4	Kyphotic_Angle	Intercept	0	[-0.194, 0.194]	1
4	Kyphotic_Angle	CVA_deg	-0.337	[-0.667, -0.007]	0.0456
4	Kyphotic_Angle	Q_Angle_deg	-0.025	[-0.232, 0.182]	0.8118
4	Kyphotic_Angle	Pelvic_Torsion	-0.143	[-0.368, 0.082]	0.2102
4	Kyphotic_Angle	Pelvic_Rotation	0.143	[-0.086, 0.372]	0.2193
4	Kyphotic_Angle	Pelvic_Drop	0.097	[-0.139, 0.333]	0.4166
4	Kyphotic_Angle	Vertebral_Rotation	-0.061	[-0.296, 0.175]	0.6107
4	Kyphotic_Angle	Lordotic_Angle	0.073	[-0.144, 0.29]	0.5065
4	Kyphotic_Angle	Sagittal_Imbalance	-0.113	[-0.329, 0.103]	0.3019
4	Kyphotic_Angle	Coronal_Imbalance	-0.099	[-0.332, 0.133]	0.3992
4	Lordotic_Angle	Intercept	0	[-0.187, 0.187]	1
4	Lordotic_Angle	CVA_deg	-0.407	[-0.72, -0.094]	0.0115
4	Lordotic_Angle	Q_Angle_deg	-0.042	[-0.242, 0.157]	0.6735
4	Lordotic_Angle	Pelvic_Torsion	0.065	[-0.153, 0.283]	0.5534
4	Lordotic_Angle	Pelvic_Rotation	0.012	[-0.21, 0.234]	0.9153
4	Lordotic_Angle	Pelvic_Drop	-0.021	[-0.248, 0.207]	0.8578
4	Lordotic_Angle	Vertebral_Rotation	-0.303	[-0.521, -0.085]	0.007
4	Lordotic_Angle	Kyphotic_Angle	0.067	[-0.134, 0.268]	0.5065
4	Lordotic_Angle	Sagittal_Imbalance	0.08	[-0.129, 0.288]	0.4506
4	Lordotic_Angle	Coronal_Imbalance	0.023	[-0.201, 0.248]	0.8362
4	Sagittal_Imbalance	Intercept	0	[-0.187, 0.187]	1
4	Sagittal_Imbalance	CVA_deg	-0.419	[-0.732, -0.106]	0.0093
4	Sagittal_Imbalance	Q_Angle_deg	-0.203	[-0.399, -0.008]	0.0412
4	Sagittal_Imbalance	Pelvic_Torsion	-0.114	[-0.331, 0.103]	0.2993
4	Sagittal_Imbalance	Pelvic_Rotation	0.108	[-0.114, 0.329]	0.3362
4	Sagittal_Imbalance	Pelvic_Drop	-0.005	[-0.234, 0.223]	0.9622
4	Sagittal_Imbalance	Vertebral_Rotation	-0.013	[-0.241, 0.214]	0.9066
4	Sagittal_Imbalance	Kyphotic_Angle	-0.105	[-0.305, 0.096]	0.3019
4	Sagittal_Imbalance	Lordotic_Angle	0.08	[-0.129, 0.289]	0.4506
4	Sagittal_Imbalance	Coronal_Imbalance	0.008	[-0.217, 0.233]	0.9439
4	Coronal_Imbalance	Intercept	0	[-0.174, 0.174]	1
4	Coronal_Imbalance	CVA_deg	-0.442	[-0.73, -0.153]	0.0031
4	Coronal_Imbalance	Q_Angle_deg	0.062	[-0.124, 0.247]	0.5096
4	Coronal_Imbalance	Pelvic_Torsion	-0.023	[-0.226, 0.181]	0.8237
4	Coronal_Imbalance	Pelvic_Rotation	-0.006	[-0.213, 0.202]	0.9573
4	Coronal_Imbalance	Pelvic_Drop	0.253	[0.047, 0.459]	0.0165
4	Coronal_Imbalance	Vertebral_Rotation	-0.144	[-0.354, 0.065]	0.1748
4	Coronal_Imbalance	Kyphotic_Angle	-0.08	[-0.267, 0.107]	0.3992
4	Coronal_Imbalance	Lordotic_Angle	0.02	[-0.175, 0.216]	0.8362
4	Coronal_Imbalance	Sagittal_Imbalance	0.007	[-0.188, 0.202]	0.9439

4	Pelvic_Drop	Intercept	0	[-0.172, 0.172]	1
4	Pelvic_Drop	CVA_deg	-0.287	[-0.579, 0.006]	0.0546
4	Pelvic_Drop	Q_Angle_deg	0.081	[-0.102, 0.263]	0.3816
4	Pelvic_Drop	Pelvic_Torsion	-0.057	[-0.257, 0.144]	0.5767
4	Pelvic_Drop	Pelvic_Rotation	0.166	[-0.035, 0.368]	0.1042
4	Pelvic_Drop	Vertebral_Rotation	-0.008	[-0.217, 0.2]	0.938
4	Pelvic_Drop	Kyphotic_Angle	0.076	[-0.109, 0.26]	0.4166
4	Pelvic_Drop	Lordotic_Angle	-0.017	[-0.21, 0.175]	0.8578
4	Pelvic_Drop	Sagittal_Imbalance	-0.005	[-0.197, 0.188]	0.9622
4	Pelvic_Drop	Coronal_Imbalance	0.246	[0.046, 0.446]	0.0165
4	Pelvic_Torsion	Intercept	0	[-0.179, 0.179]	1
4	Pelvic_Torsion	CVA_deg	-0.548	[-0.837, -0.258]	0.0003
4	Pelvic_Torsion	Q_Angle_deg	0.02	[-0.171, 0.212]	0.8321
4	Pelvic_Torsion	Pelvic_Rotation	0.016	[-0.197, 0.229]	0.883
4	Pelvic_Torsion	Pelvic_Drop	-0.062	[-0.28, 0.157]	0.5767
4	Pelvic_Torsion	Vertebral_Rotation	0.046	[-0.171, 0.264]	0.6729
4	Pelvic_Torsion	Kyphotic_Angle	-0.122	[-0.313, 0.07]	0.2102
4	Pelvic_Torsion	Lordotic_Angle	0.06	[-0.14, 0.26]	0.5534
4	Pelvic_Torsion	Sagittal_Imbalance	-0.105	[-0.304, 0.095]	0.2993
4	Pelvic_Torsion	Coronal_Imbalance	-0.024	[-0.24, 0.191]	0.8237
4	Pelvic_Rotation	Intercept	0	[-0.176, 0.176]	1
4	Pelvic_Rotation	CVA_deg	-0.288	[-0.589, 0.012]	0.0594
4	Pelvic_Rotation	Q_Angle_deg	0.009	[-0.179, 0.197]	0.9267
4	Pelvic_Rotation	Pelvic_Torsion	0.015	[-0.19, 0.221]	0.883
4	Pelvic_Rotation	Pelvic_Drop	0.175	[-0.037, 0.386]	0.1042
4	Pelvic_Rotation	Vertebral_Rotation	0.088	[-0.125, 0.302]	0.4125
4	Pelvic_Rotation	Kyphotic_Angle	0.117	[-0.071, 0.306]	0.2193
4	Pelvic_Rotation	Lordotic_Angle	0.011	[-0.187, 0.208]	0.9153
4	Pelvic_Rotation	Sagittal_Imbalance	0.095	[-0.101, 0.292]	0.3362
4	Pelvic_Rotation	Coronal_Imbalance	-0.006	[-0.217, 0.206]	0.9573
5	Vertebral_Rotation	Intercept	0	[-0.178, 0.178]	1
5	Vertebral_Rotation	CVA_deg	-0.483	[-0.757, -0.209]	0.0007
5	Vertebral_Rotation	Q_Angle_deg	0.012	[-0.175, 0.2]	0.8964
5	Vertebral_Rotation	Pelvic_Torsion	0.052	[-0.169, 0.273]	0.6404
5	Vertebral_Rotation	Pelvic_Rotation	-0.075	[-0.271, 0.122]	0.4519
5	Vertebral_Rotation	Pelvic_Drop	-0.023	[-0.243, 0.198]	0.8387
5	Vertebral_Rotation	Kyphotic_Angle	0.13	[-0.058, 0.319]	0.174
5	Vertebral_Rotation	Lordotic_Angle	0.081	[-0.119, 0.28]	0.4225
5	Vertebral_Rotation	Sagittal_Imbalance	-0.054	[-0.285, 0.176]	0.6395
5	Vertebral_Rotation	Coronal_Imbalance	-0.011	[-0.213, 0.19]	0.9134
5	Kyphotic_Angle	Intercept	0	[-0.195, 0.195]	1
5	Kyphotic_Angle	CVA_deg	-0.18	[-0.499, 0.139]	0.2649
5	Kyphotic_Angle	Q_Angle_deg	-0.055	[-0.261, 0.151]	0.5968

5	Kyphotic_Angle	Pelvic_Torsion	-0.061	[-0.304, 0.182]	0.6201
5	Kyphotic_Angle	Pelvic_Rotation	0.11	[-0.105, 0.325]	0.3139
5	Kyphotic_Angle	Pelvic_Drop	0.042	[-0.201, 0.284]	0.7331
5	Kyphotic_Angle	Vertebral_Rotation	0.157	[-0.071, 0.385]	0.174
5	Kyphotic_Angle	Lordotic_Angle	-0.071	[-0.291, 0.149]	0.5228
5	Kyphotic_Angle	Sagittal_Imbalance	-0.018	[-0.272, 0.235]	0.8863
5	Kyphotic_Angle	Coronal_Imbalance	0.119	[-0.101, 0.339]	0.2854
5	Lordotic_Angle	Intercept	0	[-0.186, 0.186]	1
5	Lordotic_Angle	CVA_deg	-0.168	[-0.472, 0.135]	0.2727
5	Lordotic_Angle	Q_Angle_deg	0.015	[-0.182, 0.211]	0.8804
5	Lordotic_Angle	Pelvic_Torsion	-0.124	[-0.354, 0.106]	0.2853
5	Lordotic_Angle	Pelvic_Rotation	0.095	[-0.109, 0.3]	0.3576
5	Lordotic_Angle	Pelvic_Drop	0.171	[-0.057, 0.399]	0.1392
5	Lordotic_Angle	Vertebral_Rotation	0.088	[-0.13, 0.307]	0.4225
5	Lordotic_Angle	Kyphotic_Angle	-0.064	[-0.263, 0.135]	0.5228
5	Lordotic_Angle	Sagittal_Imbalance	0.242	[0.007, 0.478]	0.044
5	Lordotic_Angle	Coronal_Imbalance	-0.116	[-0.325, 0.093]	0.2735
5	Sagittal_Imbalance	Intercept	0	[-0.162, 0.162]	1
5	Sagittal_Imbalance	CVA_deg	-0.358	[-0.613, -0.104]	0.0063
5	Sagittal_Imbalance	Q_Angle_deg	0.116	[-0.053, 0.285]	0.177
5	Sagittal_Imbalance	Pelvic_Torsion	-0.042	[-0.242, 0.159]	0.6818
5	Sagittal_Imbalance	Pelvic_Rotation	0.029	[-0.15, 0.208]	0.7488
5	Sagittal_Imbalance	Pelvic_Drop	0.098	[-0.101, 0.298]	0.3313
5	Sagittal_Imbalance	Vertebral_Rotation	-0.045	[-0.235, 0.145]	0.6395
5	Sagittal_Imbalance	Kyphotic_Angle	-0.012	[-0.186, 0.161]	0.8863
5	Sagittal_Imbalance	Lordotic_Angle	0.183	[0.005, 0.361]	0.044
5	Sagittal_Imbalance	Coronal_Imbalance	0.24	[0.064, 0.416]	0.0081
5	Coronal_Imbalance	Intercept	0	[-0.185, 0.185]	1
5	Coronal_Imbalance	CVA_deg	-0.24	[-0.539, 0.059]	0.1144
5	Coronal_Imbalance	Q_Angle_deg	-0.052	[-0.247, 0.143]	0.5951
5	Coronal_Imbalance	Pelvic_Torsion	0.007	[-0.223, 0.237]	0.9502
5	Coronal_Imbalance	Pelvic_Rotation	0.041	[-0.163, 0.246]	0.6878
5	Coronal_Imbalance	Pelvic_Drop	-0.06	[-0.289, 0.169]	0.6041
5	Coronal_Imbalance	Vertebral_Rotation	-0.012	[-0.23, 0.206]	0.9134
5	Coronal_Imbalance	Kyphotic_Angle	0.106	[-0.09, 0.303]	0.2854
5	Coronal_Imbalance	Lordotic_Angle	-0.115	[-0.321, 0.092]	0.2735
5	Coronal_Imbalance	Sagittal_Imbalance	0.314	[0.084, 0.544]	0.0081
5	Pelvic_Drop	Intercept	0	[-0.169, 0.169]	1
5	Pelvic_Drop	CVA_deg	-0.395	[-0.659, -0.13]	0.0038
5	Pelvic_Drop	Q_Angle_deg	-0.077	[-0.255, 0.1]	0.3902
5	Pelvic_Drop	Pelvic_Torsion	0.193	[-0.013, 0.399]	0.0654
5	Pelvic_Drop	Pelvic_Rotation	-0.058	[-0.244, 0.128]	0.5366
5	Pelvic_Drop	Vertebral_Rotation	-0.02	[-0.219, 0.178]	0.8387

5	Pelvic_Drop	Kyphotic_Angle	0.031	[-0.149, 0.212]	0.7331
5	Pelvic_Drop	Lordotic_Angle	0.141	[-0.047, 0.328]	0.1392
5	Pelvic_Drop	Sagittal_Imbalance	0.107	[-0.111, 0.324]	0.3313
5	Pelvic_Drop	Coronal_Imbalance	-0.05	[-0.241, 0.141]	0.6041
5	Pelvic_Torsion	Intercept	0	[-0.168, 0.168]	1
5	Pelvic_Torsion	CVA_deg	-0.378	[-0.642, -0.113]	0.0057
5	Pelvic_Torsion	Q_Angle_deg	0.143	[-0.032, 0.318]	0.108
5	Pelvic_Torsion	Pelvic_Rotation	0.176	[-0.007, 0.358]	0.0589
5	Pelvic_Torsion	Pelvic_Drop	0.193	[-0.013, 0.398]	0.0654
5	Pelvic_Torsion	Vertebral_Rotation	0.047	[-0.151, 0.245]	0.6404
5	Pelvic_Torsion	Kyphotic_Angle	-0.045	[-0.225, 0.135]	0.6201
5	Pelvic_Torsion	Lordotic_Angle	-0.102	[-0.29, 0.086]	0.2853
5	Pelvic_Torsion	Sagittal_Imbalance	-0.045	[-0.263, 0.173]	0.6818
5	Pelvic_Torsion	Coronal_Imbalance	0.006	[-0.185, 0.197]	0.9502
5	Pelvic_Rotation	Intercept	0	[-0.189, 0.189]	1
5	Pelvic_Rotation	CVA_deg	-0.226	[-0.533, 0.081]	0.1464
5	Pelvic_Rotation	Q_Angle_deg	-0.014	[-0.213, 0.186]	0.8931
5	Pelvic_Rotation	Pelvic_Torsion	0.222	[-0.009, 0.453]	0.0589
5	Pelvic_Rotation	Pelvic_Drop	-0.073	[-0.308, 0.161]	0.5366
5	Pelvic_Rotation	Vertebral_Rotation	-0.084	[-0.307, 0.138]	0.4519
5	Pelvic_Rotation	Kyphotic_Angle	0.103	[-0.099, 0.304]	0.3139
5	Pelvic_Rotation	Lordotic_Angle	0.099	[-0.113, 0.311]	0.3576
5	Pelvic_Rotation	Sagittal_Imbalance	0.04	[-0.206, 0.285]	0.7488
5	Pelvic_Rotation	Coronal_Imbalance	0.043	[-0.171, 0.258]	0.6878
